# Real-world clinical and molecular management of 50 prospective patients with microphthalmia, anophthalmia and/or ocular coloboma

**DOI:** 10.1136/bjo-2022-321991

**Published:** 2022-10-03

**Authors:** Philippa Harding, Sri Gore, Samantha Malka, Jayashree Rajkumar, Ngozi Oluonye, Mariya Moosajee

**Affiliations:** 1 Institute of Ophthalmology, University College London, London, UK; 2 Moorfields Eye Hospital NHS Foundation Trust, London, UK; 3 Great Ormond Street Hospital For Children NHS Trust, London, UK

**Keywords:** Genetics, Embryology and development, Eye (Globe), Vision

## Abstract

**Background/aims:**

Microphthalmia, anophthalmia and coloboma (MAC) are clinically and genetically heterogenous rare developmental eye conditions, which contribute to a significant proportion of childhood blindness worldwide. Clear understanding of MAC aetiology and comorbidities is essential to providing patients with appropriate care. However, current management is unstandardised and molecular diagnostic rates remain low, particularly in those with unilateral presentation. To further understanding of clinical and genetic management of patients with MAC, we charted their real-world experience to ascertain optimal management pathways and yield from molecular analysis.

**Methods:**

A prospective cohort study of consecutive patients with MAC referred to the ocular genetics service at Moorfields Eye Hospital between 2017–2020.

**Results:**

Clinical analysis of 50 MAC patients (15 microphthalmia; 2 anophthalmia; 11 coloboma; and 22 mixed) from 44 unrelated families found 44% had additional ocular features (complex) and 34% had systemic involvement, most frequently intellectual/developmental delay (8/17). Molecular analysis of 39 families using targeted gene panels, whole genome sequencing and microarray comparative genomic hybridisation identified genetic causes in, 28% including novel variants in six known MAC genes (*SOX2*, *KMT2D*, *MAB21L2*, *ALDH1A3*, *BCOR* and *FOXE3*), and a molecular diagnostic rate of 33% for both bilateral and unilateral cohorts. New phenotypic associations were found for *FOXE3* (bilateral sensorineural hearing loss) and *MAB21L2* (unilateral microphthalmia).

**Conclusion:**

This study highlights the importance of thorough clinical and molecular phenotyping of MAC patients to provide appropriate multidisciplinary care. Routine genetic testing for both unilateral and bilateral cases in the clinic may increase diagnostic rates in the future, helping elucidate genotype–phenotype correlations and informing genetic counselling.

**What is already known on this topic**

- Microphthalmia, anophthalmia and coloboma (MAC) is a clinically and genetically heterogeneous group of rare developmental conditions, with low molecular diagnostic rates and few established genotype–phenotype correlations. Management of patients is highly variable and requires multidisciplinary care teams, including clinical scientists, geneticists, ophthalmologists and genetic counsellors.

**What this study adds**

- Here we describe detailed clinical phenotyping of 50 prospective MAC patients to investigate trends in a heterogeneous cohort: 22/50 (44%) had complex ocular features and 17/50 (34%) displayed systemic manifestations, the most prevalent being developmental delay/intellectual disability (8/17, 47%). Abnormal brain MRIs were more frequently associated with bilateral MAC. Molecular testing discovered identified a genetic association in 11/39 families (28%), including novel variants of known MAC genes *SOX2*, *KMT2D*, *MAB21L2*, *ALDH1A3*, and *BCOR* and *FOXE3* with expansion of the disease phenotype associated with *MAB21L2* and *FOXE3*.

**How this study might affect research, practice or policy**

- Over one-third of patients have systemic associations requiring multidisciplinary input. An increase in genetic diagnoses aids with informed genetic counselling, family planning and disease prognosis. Detailed clinical phenotyping and molecular testing revealed ocular and systemic trends in a typical MAC cohort, including novel phenotypic associations and pathogenic variants, important for appropriate clinical care and genetic counselling.

## Introduction

Microphthalmia, anophthalmia and ocular coloboma (MAC) fall within the same phenotypic spectrum of ocular maldevelopment. Anophthalmia is defined as no visible ocular globe, while microphthalmia consists of a small eye (axial length ≥2 SD below the normal axial length), both of which are caused by disrupted eye development, resulting in failure to initiate or premature arrest of oculogenesis.^1^ Ocular coloboma is a persistent inferonasal tissue defect arising from incomplete fusion of the optic fissure, affecting one or more structures including the iris, ciliary body and retina.^2^ MAC may present in one or both eyes (unilateral/bilateral) and can occur in combination (mixed) within the same eye or contralateral eyes. MAC can be simplex, with no additional ocular complications, but frequently manifests with complex associated features including cataracts and anterior segment dysgenesis.^1^ Furthermore, between 33% and 95% of patients display extraocular involvement (syndromic).^3 4^ MAC has a combined prevalence of between 1 and 4 live births per 10 000^4 5^ and often results in reduced visual acuity, contributing up to 15% of childhood blindness and severe visual impairment worldwide.^6^

Genetic or environmental factors disturbing eye development from 3 weeks of gestation can cause MAC by altering molecular signalling pathways regulating oculogenesis.^1 4 7^ Over 100 monogenic associations with MAC have been identified as well as large chromosomal anomalies detected in up to 15% of patients through conventional cytogenetics,^5 7 8^ although lower rates are reported in non-syndromic cohorts (8%).^9^ Clinical heterogeneity in MAC cohorts is partially due to the spectrum of causative genes playing diverse roles in controlling multiorgan/tissue development. Discovering genotype–phenotype correlations can guide patient management and genetic counselling.^3 10^ While a genetic cause can be identified in 80% of bilateral severe microphthalmia and anophthalmia patients, only 10% of unilateral patients typically receive a molecular diagnosis.^7^ Improving molecular diagnostic rates through increased genetic testing and identification of novel variants will improve understanding of genotype–phenotype relationships.

This study reports clinical evaluation and genetic testing results of 50 MAC patients referred to Moorfields Eye Hospital (MEH) NHS Foundation Trust ocular genetics service prospectively over a 29-month period.

## Materials and methods

### Editorial policies and ethical considerations

This study had relevant local and national research ethics committee approvals (MEH and the Northwest London Research Ethics Committee) and adhered to the tenets of the Declaration of Helsinki. Patients and relatives gave written informed consent for participation through either the Genetic Study of Inherited Eye Disease (REC reference 12/LO/0141, 10 October 2016) or Genomics England 100 000 Genomes project (REC reference 14/EE/1112, 20 February 2015).

### Clinical evaluation

Patients with microphthalmia (axial length <16 mm at birth, <19 mm at 12 months, <21 mm in adult eyes), anophthalmia (no evidence of a globe or ocular tissue in the orbit on clinical examination/MRI scan) and/or ocular coloboma presenting to the ocular genetics service at MEH between 1 September 2017 and 30 January 2020 were included in this study. Patients were classed as simplex MAC if no other additional ocular phenotype was seen; mixed MAC was a combination of two or more MAC features in the same or contralateral eye; complex MAC was considered the presence any non-MAC ocular features in the MAC-affected or contralateral eye. Patients were classed as syndromic if any non-ocular phenotype was identified or non-syndromic is no systemic/extraocular involvement. Each patient underwent detailed clinical evaluation, including full history, orthoptic assessment, refraction, best-corrected visual acuity (BCVA) measured using LogMAR or Cardiff cards for preverbal children up to 36 months of age where possible; slit lamp examination and fundus examination were recorded with anterior segment imaging using the Haag-Streit slit lamp camera (Haag-Streit Holdings AG, Köniz, Switzerland) and ultra-widefield fundus colour imaging with the Optos California. Further investigations included orbital ultrasound to measure axial length, electrophysiology and MRI brain and orbits (where applicable). Every patient was evaluated by a paediatrician for systemic associations.

### Patients and genetic analysis

Whole genome sequencing (WGS) analysis was undertaken through the 100 000 Genomes Project.^11^ Genomic DNA was processed using Illumina TruSeq DNA PCR-Free Sample Preparation kits (Illumina) and sequenced using the Illumina HiSeq X Ten high-throughput sequencing platform, generating minimum coverage of 15X for >97% of the callable autosomal genome.^12^ Readings were aligned to build GRCh37/GRCh38 of the human genome using an Isaac aligner (Illumina Inc). Single-nucleotide variants and indels were identified using Platypus software (V.0.8.1) and annotated using Cellbase (https://github.com/opencb/cellbase). Variant filtering was performed using minor allele frequency <0.001 in publicly available and in-house data sets, predicted protein effect and familial segregation. Surviving variants were prioritised using the ‘microphthalmia or anophthalmia’ or ‘ocular coloboma’ virtual gene panels (https://panelapp.genomicsengland.co.uk/panels/). WGS data were not analysed for copy number variants (CNVs) through an automated pipeline; however, CNVs were investigated on a case-by-case basis.

Targeted gene panel testing of MAC genes was conducted through the Rare & Inherited Disease Genomic Laboratory at Great Ormond Street Hospital for Children (GOSH, London, UK). Variant screening was carried out by library preparation using Agilent-focused clinical exome+1 kit followed by next-generation sequencing on Illumina platforms. Data were analysed using in-house pipelines and virtual gene panels, with variants confirmed by Sanger sequencing. Larger insertion/deletion mutations and CNVs are routinely investigated using ExomeDepth, but the sensitivity to detect these mutation types is unknown. aCGH, which is able to detect pathogenic CNVs, was performed through GOSH, together with segregation of known familial variants as previously described.^12^

Literature searches along with public databases HGMD (http://www.hgmd.cf.ac.uk),^13^ Clinvar (https://www.ncbi.nlm.nih.gov/clinvar/)^14^ and gnomAD^15^ were examined for prior reports of variants found in this cohort. Likely pathogenicity of novel variants was assessed using the predictive algorithms gnomAD, PolyPhen-2,^16^ SIFT^17^ and PredictSNP.^18^ Genetic results were reviewed by a multidisciplinary team (including clinical scientists, specialists in clinical genetics and ophthalmology), to confirm variant pathogenicity, prevalence in publicly available genome databases, the clinical phenotype and mode of inheritance, before the molecular diagnosis was established. Novel variants in this study were submitted to ClinVar.

### Statistical testing

Statistical tests were performed in GraphPad Prism V.8.2.1 (www.graphpad.com).

## Results

### Patient demographics

**Figure 1:**
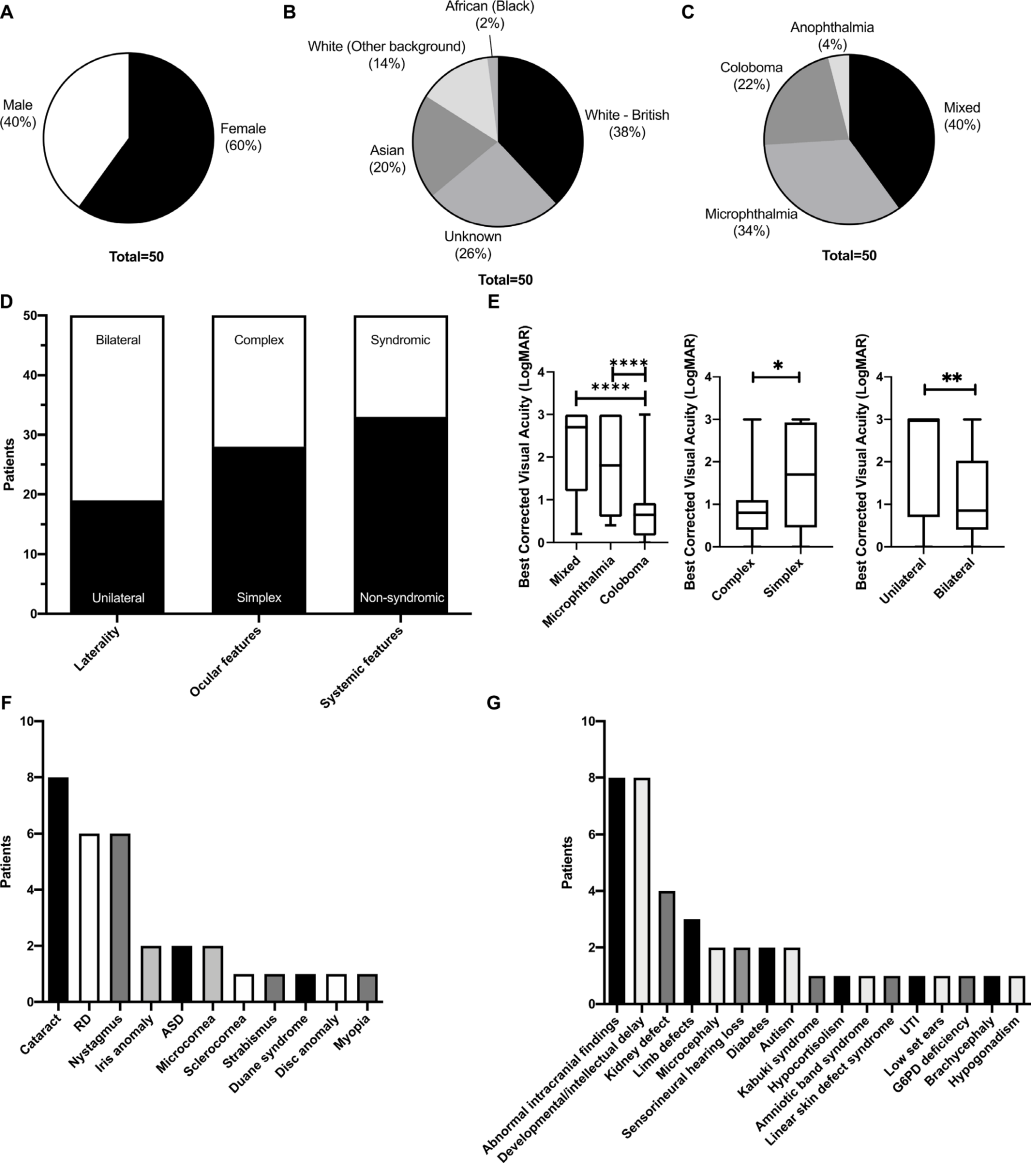
Demographics and clinical phenotype of 50 microphthalmia, anophthalmia and ocular coloboma (MAC) patients: (a) Sex; (b) Ethnicity; (c) MAC phenotype of patients divided into exclusively microphthalmia/anophthalmia/ocular coloboma or a combination of MAC (mixed); (d) Ocular and systemic features of MAC cohort: laterality (unilateral/bilateral MAC); presence of associated non-MAC ocular phenotype in affected or contralateral eye (simplex/complex); presence of systemic features (syndromic/non-syndromic). (e) Mean best corrected visual acuities of MAC-affected eyes; (f) Additional ocular phenotypes present in MAC patients; (g) Extraocular associations in MAC patients (abnormal intracranial findings refer to significant findings which would affect the health of the patient, including agenesis of corpus callosum, hydrocephalus and cerebellar hypoplasia; developmental delay includes intellectual/learning difficulties and global developmental delay. ASD, anterior segment dysgenesis; G6P6, glucose-6-phosphate dehydrogenase; RD, retinal detachment; UTI, urinary tract infections.

Fifty patients with MAC from 44 families with a mean age of 13 years (1-month 64 years) were referred to the ocular genetics service at MEH (table 1). Sixty per cent were women (30/50) and ethnicity encompassed 19 white (British, 38%), 10 Asian (20%), 7 white (other background, 14%), 1 black (African, 2%) and 13 unknown (26%) (figure 1A,B).

**Table 1:** Clinical phenotype and molecular diagnosis of 50 microphthalmia, anophthalmia and coloboma (MAC) patients

Family: patient ID (GC number)	Ethnicity	Sex	Age range (at referral)	Unilateral/ bilateral	MAC phenotype		Simplex/ complex	Associated ocular features	Visual acuity		Prosthesis		Syndromic/ Non-syndromic	Systemic features	Molecular test	Genetic diagnosis	Inheritance
					Right	Left			Right	Left	Right	Left					
1–1 (15781)	Asian—Pakistani	F	20–29	Bilateral	Microphthalmia, iris and retinal coloboma	Iris and retinal coloboma	Complex	Right convergent strabismus, nystagmus	1.80	0.18	CCS	–	Syndromic	G6PD deficiency	WGS	–	Unknown (simplex)
2–1 (25618)	White—British	M	60–69	Bilateral	Anophthalmia	Coloboma	Simplex	–	Unk.	1.00	CCP	–	Syndromic	DM type 2	WGS	–	Unknown (simplex)
3–1 (12652)	White—British	F	30–39	Unilateral	Microphthalmia	–	Simplex	–	3.00	−0.08	CCP	–	Non-syndromic	–	WGS	–	Suspected AD (affected mother)
4–1 (14100)	White—Other background	F	40–49	Bilateral	Microphthalmia, chorioretinal coloboma	Chorioretinal coloboma (macula-sparing)	Complex	Right RD	3.00	0.60	–	–	Non-syndromic	–	aCGH	Chr11 large deletion (including *YAP1*)	AD (de novo)
5–1 (20618)	White—British	F	30–39	Bilateral	Microphthalmia, optic disc coloboma	Microphthalmia, optic disc coloboma	Simplex	–	Unk.	Unk.	–	–	Non-syndromic	–	WGS	–	Unknown (simplex)
6–1 (26614)	Unknown	M	30–39	Bilateral	Anophthalmia	Anophthalmia	Simplex	–	Unk.	Unk.	–	–	Syndromic	Hypogonadism	aCGH, WES-Oculome panel	*SOX2*	AD (simplex; likely de novo)
7–1 (14206)	White—British	F	30–39	Unilateral	–	Microphthalmia, optic nerve coloboma	Complex	Right aniridia, right aphakia-	1.00	3.00	–	CCP	Syndromic	DM type 2	Single gene (*PAX6*)	*PAX6*	AD (affected relatives)
8–1 (25830)	White—British	M	50–59	Bilateral	Optic disc coloboma	Microphthalmia, iris coloboma	Complex	Left microcornea, left cataract, right RD	1.00	2.70	–	–	Non-syndromic	–	WGS	–	Unknown (simplex)
9–1 (25409)	Unknown	M	0–9	Unilateral	–	Microphthalmia, iris and chorioretinal coloboma	Simplex	–	0.80	3.00	–	–	Syndromic	Kabuki syndrome, submucosal cleft palate	WES-Oculome panel	*KMT2D*	AD (simplex; likely de novo)
10–1 (29326)	White—British	F	10–19	Bilateral	Chorioretinal coloboma	Chorioretinal coloboma	Complex	Bilateral RDs and vitrectomy	3.00	1.20	–	–	Non-syndromic	–	–	–	Unknown (simplex)
11–1 (25470)	Unknown	M	0–9	Unilateral	–	Microphthalmia, iris and chorioretinal coloboma	Simplex	–	0.60	Unk.	–	–	Syndromic	Amniotic band syndrome (multiple distill limb anomalies)	–	–	Unknown (simplex)
12–1 (25531)	Unknown	F	0–9	Unilateral		Microphthalmia, disc coloboma	Complex	Left RD	0.30	Unk.	–	CCP	Non-syndromic	–	WGS	–	Unknown (simplex)
13–1 (25529)	White—British	F	10–19	Unilateral	–	Microphthalmia	Simplex	–	0.00	3.00	–	CCP	Non-syndromic	–	WGS	–	Unknown (simplex)
14–1 (29327)	Asian—Bengali	F	10–19	Bilateral	Microphthalmia	Microphthalmia	Complex	Corneal opacity	2.70	2.70	–	–	Syndromic	Linear skin defect syndrome, intellectual/developmental disability, abnormal intracranial findings) (hydrocephalous, agenesis of corpus callosum, cerebellar hypoplasia)	aCGH	ChrX large deletion mosaic	XLD (simplex; mosaicism)
15–1 (15190)	White—British	M	10–19	Bilateral	Microphthalmia	Microphthalmia	Complex	Congenital cataract, nystagmus, aphakia	0.40	1.40	–	–	Non-syndromic	–	WGS	*EPHA2*	AD (strong family history)
15–2 (15190)	White—British	M	0–9	Bilateral	Microphthalmia	Microphthalmia	Complex	Congenital cataract, nystagmus, aphakia	0.60	0.70	–	–	Non-syndromic	–	WGS	*EPHA2*	AD (strong family history)
15–3 (15190)	White—British	M	10–19	Bilateral	Microphthalmia	Microphthalmia	Complex	Congenital cataract, nystagmus, aphakia	0.40	0.40	–	–	Non-syndromic	–	WGS	*EPHA2*	AD (strong family history)
15–4 (15190)	White—British	F	40–49	Bilateral	Microphthalmia	Microphthalmia	Complex	Congenital cataract, nystagmus, aphakia, glaucoma	1.00	0.80	–	–	Non-syndromic	–	WGS	*EPHA2*	AD (strong family history)
16–1 (25756)	Asian—Indian	F	0–9	Unilateral	Microphthalmia	–	Simplex	–	3.00	0.08	–	–	Non-syndromic	–	WGS	*MAB21L2*	AD (simplex; de novo or mosaicism)
17–1 (25769)	White—British	M	0–9	Unilateral	Microphthalmia, chorioretinal coloboma (macula-sparing)	–	Complex	Right RD	3.00	0.02	CCS	–	Non-syndromic	–	–	–	Unknown (simplex)
18–1 (25841)	Unknown	F	0–9	Bilateral	Microphthalmia, iris and chorioretinal coloboma	Chorioretinal coloboma	Complex	Hypermetropia	0.30	0.20	–	–	Non-syndromic	–	WES-Oculome panel	–	Unknown (simplex)
19–1 (25854)	White—Slovakian	M	0–9	Unilateral	Chorioretinal coloboma	–	Complex	Right atypical iris polycoria	1.00	0.00	–	–	Syndromic	Hypocortisolism	WGS	–	Unknown (simplex)
20–1 (25198)	White—Other background	F	0–9	Bilateral	Microphthalmia, chorioretinal coloboma	Chorioretinal coloboma	Simplex	–	0.20	0.20	–	–	Non-syndromic	–	WGS	–	Unknown (affected sibling)
20–2	White—Other background	F	0–9	Bilateral	Iris, chorioretinal and optic nerve coloboma	Iris and chorioretinal coloboma	Complex	Myopia	0.8	0.8	–	–	Non-syndromic	–	WGS	–	Unknown (affected sibling)
21–1 (25890)	White—Other background	M	0–9	Unilateral	–	Microphthalmia	Simplex	–	0.00	3.00	–	CCP	Syndromic	Learning disabilities	WGS	–	Unknown (simplex)
22–1 (25966)	Asian—Pakistani	F	0–9	Bilateral	Anophthalmia	Anophthalmia	Simplex	–	Unk.	Unk.	CCP	CCP	Syndromic	Autism spectrum, intellectual disability-	WGS	*ALDH1A3*	AR (simplex; consanguinity)
23–1 (26133)	Black—Somalian	M	0–9	Unilateral	–	Microphthalmia	Complex	Right small lens opacity	0.20	Unk.	–	CCP	Non-syndromic	–	WGS	–	Unknown (simplex)
24–1 (26134)	White—Other background	F	0–9	Bilateral	Microphthalmia, optic disc coloboma	Microphthalmia, optic disc coloboma	Simplex	–	3.00	3.00	CCP	CCP	Syndromic	Hydronephrosis, one kidney larger than other (left duplex, right dysplastic), multiple UTI, single palmar crease left hand, low set ears, developmental delay, brachycephaly, severe microcephaly, abnormal intracranial findings (simple gyral pattern, cerebellar hypoplasia, short corpus callosum and post positioned pituitary)	WGS	–	Unknown (simplex)
25–1 (26152)	White—British	F	0–9	Bilateral	Microphthalmia, iris and chorioretinal coloboma	Microphthalmia, iris and chorioretinal coloboma	Simplex	–	Unk.	2.70	–	–	Syndromic	Right kidney scarring, recurrent UTI	WGS	–	Unknown (simplex)
26–1 (27120)	White—Spanish	M	0–9	Unilateral	Microphthalmia, chorioretinal coloboma (macula-sparing)	–	Complex	Right RD	Unk.	0.60	CCP	–	Syndromic	Bilateral webbed toes	–	–	Unknown (simplex)
27–1 (29328)	Unknown	M	0–9	Unilateral	Iris and inferior chorioretinal coloboma	–	Simplex	–	0.30	0.30	–	–	Non-syndromic	–	–	–	Unknown (simplex)
28–1 (23067)	White—British	F	10–19	Unilateral	–	Microphthalmia	Complex	Bilateral congenital cataracts, aphakia glaucoma	2.70	0.60	–	–	Non-syndromic	–	WGS	*BCOR*	XLD (de novo)
29–1 (26308)	Asian—Indian	F	0–9	Bilateral	Microphthalmia, posterior and anterior coloboma	Posterior coloboma	Complex	Right congenital cataracts	Unk.	Unk.	–	–	Non-syndromic	–	WES-Oculome panel	–	Unknown (simplex)
30–1 (21852)	White—British	F	0–9	Unilateral	Microphthalmia	–	Simplex	–	3.00	0.22	CCS	–	Non-syndromic	–	aCGH	Chr10 deletion	AD (de novo)
31–1 (26385)	White—British	M	10–19	Unilateral	Optic disc and retinal coloboma	–	Complex	Bilateral anomalous optic disc	0.00	0.00	–	–	Non-syndromic	–	WES-Oculome panel	–	Suspected AD (affected father)
32–1 (26461)	Unknown	F	0—9	Bilateral	Iris coloboma	Iris coloboma	Simplex	–	0.10	0.10	–	–	Non-syndromic	–	WES-Oculome panel	–	Unknown (simplex)
33–1 (26484)	Asian—Bangladeshi	F	0–9	Bilateral	Chorioretinal coloboma	Chorioretinal coloboma	Simplex	–	0.50	1.60	–	–	Syndromic	Microcephaly, abnormal intracranial findings (vascular anomaly of the scalp), mild developmental delay/intellectual disability, autism	WES-Oculome panel	–	Unknown (simplex; consanguinity)
34–1 (27784)	White—British	F	0–9	Bilateral	Microphthalmia	Microphthalmia	Simplex	–	Unk.	Unk.	CCS	CCS	Syndromic	Right multi cystic dysplastic kidney, abnormal intracranial findings (congenital frontal lobe cyst, multiple paraventricular cysts, hypoplastic corpus callosum, incomplete vestibular turns)	WES-Oculome panel	–	Unknown (simplex)
35–1 (28266)	Asian—Syrian	M	0–9	Bilateral	Microphthalmia	Microphthalmia	Simplex	–	Unk.	Unk.	CCS	CCS	Syndromic	Bilateral sensorineural hearing loss, global developmental delay	Single gene (*FOXE3*)	*FOXE3*	AR (affected sibling)
35–2 (28266)	Asian—Syrian	F	0–9	Bilateral	Microphthalmia	Microphthalmia	Simplex	–	2.70	2.70	CCS	CCS	Syndromic	Bilateral enlarged kidneys with multiple cysts, bilateral sensorineural hearing loss, severe intellectual disability	WES-Oculome panel	*FOXE3*	AR (affected sibling)
36–1 (26877)	Asian—Bangladeshi	F	0–9	Bilateral	Iris and chorioretinal coloboma	Microphthalmia, iris and chorioretinal coloboma	Simplex	–	Unk.	Unk.	–	–	Non-syndromic	–	WES-Oculome panel	–	Unknown (simplex)
37–1 (27046)	Asian—Iraqi Kurdish	F	10–19	Bilateral	Microphthalmia	Optic disc and chorioretinal coloboma	Simplex	–	1.80	0.00	CCS	–	Non-syndromic	–	WES-Oculome panel	–	Unknown (simplex)
38–1 (27094)	Unknown	M	0–9	Bilateral	Iris and chorioretinal coloboma	Iris and chorioretinal coloboma	Simplex	–	0.08	0.44	–	–	Non-syndromic	–	WES-Oculome panel	–	Unknown (simplex)
39–1 (27259)	Unknown	F	0–9	Bilateral	Optic disc coloboma	Microphthalmia, chorioretinal coloboma involving disc	Simplex	–	0.70	0.70	–	–	Non-syndromic	Abnormal intracranial findings (ipsilateral optic nerve hypoplasia, hemichiasm, pituitary gland hypoplasia, large orbital cyst)	WES-Oculome panel	–	Unknown (simplex)
40–1 (26959)	White—British	M	50–59	Unilateral	Microphthalmia iris and chorioretinal coloboma	–	Simplex	–	2.20	0.00	–	–	Non-syndromic	–	WES-Oculome panel	–	Suspected AD (affected son)
40–2 (26959)	White—British	M	10–19	Bilateral	Microphthalmia, iris and chorioretinal coloboma	Microphthalmia, iris and chorioretinal coloboma	Simplex	–	2.70	1.00	–	–	Non-syndromic	–	WES-Oculome panel	–	Suspected AD (affected father)
41–1 (27355)	Unknown	F	0–9	Bilateral	Optic disc coloboma	Optic disc coloboma	Simplex	–	0.90	0.90	–	–	Non-syndromic	–	WES-Oculome panel	–	Unknown (simplex)
42–1 (27454)	Unknown	M	0–9	Bilateral	Iris and chorioretinal coloboma	Optic disc coloboma	Complex	Left duanes	0.10	0.90	–	–	Non-syndromic	Abnormal intracranial findings (hypoplasia of the abducens nerve)	WES-Oculome panel	–	Suspected AD (affected father)
43–1 (27605)	Unknown	F	0–9	Unilateral	–	Microphthalmia, optic disc coloboma	Simplex	–	0.10	2.70	–	–	Non-syndromic	Abnormal intracranial findings (ipsilateral optic nerve hypoplasia, hemichiasm)	WES-Oculome panel	–	Unknown (simplex)
44–1 (27631)	Unknown	F	0–9	Unilateral	–	Uveal iris coloboma	Complex	Right inferior iris anomaly	1.20	0.70	–	–	Non-syndromic	–	WES-Oculome panel	–	Unknown (simplex)

Oculome panel – http://www.labs.gosh.nhs.uk/media/764794/oculome_v8.pdf

aCGH, microarray comparative genomic hybridisation; AD, autosomal dominant; AR, autosomal recessive; CCP, customised cosmetic prosthesis; CCS, customised clear shell; Chr, chromosome; DM, diabetes mellitus; F, female; GC number, genetic counselling number; G6P6, glucose-6-phosphate dehydrogenase; M, male; RD, retinal detachment; Unk, Unknown; UTI, urinary tract infection; WES, whole exome sequencing; WGS, whole genome sequencing; XLD, X-linked dominant.

Of the 44 families, 36 were simplex cases with no known family history (82%), 2 had affected siblings only (5%) and 6 had a multi-generational family history (14%). Two cases were known to be the offspring of consanguineous parents (5%).

### Ocular phenotype

Of the cohort, 22/50 exhibited a combination of MAC phenotypes in one or both eyes (mixed, 44%) while 15/50 had only microphthalmia (30%), 2/50 anophthalmia (4%) and 11/50 coloboma (22%) (figure 1C). Sixty-two per cent had bilateral (31/50), while 38% had unilateral (19/50) involvement. Twenty-eight patients (56%) had simplex MAC of which 10/28 were unilateral (36%), and 43% (12/28) had simplex MAC but with syndromic features (43%) (figure 1D). Twenty-two patients had at least one non-MAC-associated ocular abnormality in the affected or contralateral eye (44%) (complex phenotype), the most common of which was cataracts (8/50, 16%) followed by nystagmus (6/50, 12%) (figure 1F). Of the complex patients, 9/22 were unilateral (41%), and 5/22 (23%) had syndromic features. The most prevalent complex MAC condition was microphthalmia, with 8/22 patients presenting with only microphthalmia (37%), 6/22 with only coloboma (27%) and 8 with a mixed combination of microphthalmia and coloboma (37%).

Mean BCVA of all MAC affected eyes which had a recorded visual acuity (61/81) was 1.4 LogMAR (0.00–3.00) (table 1). Coloboma-affected eyes had a mean BCVA of 0.667 LogMAR (0.00–3.00), microphthalmic eyes of 1.78 LogMAR (0.40–3.00) and mixed MAC eyes of 2.18 LogMAR (0.20–3.00) (figure 1E). Anophthalmia eyes had no measurable visual acuity. T tests revealed significantly better BCVA in coloboma-affected eyes compared with microphthalmic eyes (p<0.0001), and those with a mixed MAC phenotype (p<0.0001). T tests revealed significantly better visual acuity in eyes affected with any additional ocular features (p=0.023), which may be due to complex features such as cataracts or nystagmus co-occurring more often with coloboma than microphthalmia/anophthalmia in this cohort, with 6/21 (29%) complex MAC-affected eyes exhibiting only coloboma (without anophthalmia/microphthalmia) compared with 5/29 simplex eyes (17%). Unilaterally affected eyes had significantly worse BCVA than bilateral patients (p=0.005), although again may be biased by the specific MAC phenotype, such as a higher proportion of microphthalmic/anophthalmic unilateral affected eyes (15/19–79%) compared with bilateral eyes (38/62–61%). Associated amblyopia may also have contributed to worse BCVA in unilateral patients.

Of the eight cataract patients, seven had lensectomy and were left aphakic. The age of surgical intervention ranged from 6 weeks to 28 years. Two eyes developed secondary glaucoma following lensectomy; there were no primary cases of glaucoma in this series.

Retinal detachments (RDs) were observed in seven affected eyes from six patients (7/80, 9%) all with ocular coloboma±microphthalmia. Six patients had unilateral RD; patient 10–1 had bilateral consecutive RDs: patient 10–1 had symptoms in one eye, but the other eye was found to have a recent onset RD in the other better-seeing eye during time of examination under anaesthesia. The detachments were thought to have occurred within several months of each other, but no trauma had been reported. No molecular tests were performed for this patient. Three eyes had only coloboma (8–1 had optic disc; 10–1 had bilateral chorioretinal) while four had coloboma with microphthalmia (12–1 had optic disc; 4–1, 17–1 and 26.1 had macula-sparing chorioretinal). RDs occurred before the age of 1 year in three eyes (12–1, 17–1, 26–1). Vitreoretinal surgery was performed on patient 10–1 as both eyes had visual potential, but other patients were treated conservatively due to the extent of the detachment at presentation or poor visual potential. Unoperated eyes became painless pthtisical eyes (5/7).

The majority of patients did not need or were unsuitable for prosthetic rehabilitation because of normal or near-normal axial lengths (31/50, 62%). Of the 19/50 who were candidates for prosthetics, 2/19 (11%) did not undergo prosthetic rehabilitation despite low axial lengths (patient 6–1 decided not to pursue prosthetics and patient 39–1 could not retain a prosthesis due to presence of a developmental cyst). Nineteen microphthalmic eyes underwent prosthetic rehabilitation, along with three anophthalmic eyes. Patients who underwent prosthetic rehabilitation had bespoke treatment plans depending on factors such as age of presentation, size of affected eye and patient/parent request (table 1): 10 customised cosmetic prosthesis (CCP), 7 customised clear shell (CCS). All eyes fitted with CCS were microphthalmic (7/7), while 75% CCP eyes were microphthalmic (9/12) with the remainder being anophthalmic (3/12).

### Systemic features

Systemic associations were found in 17/50 patients (34%). The most common systemic features were developmental delay/intellectual disability (8/17, 47%) followed by kidney defects (4/17, 24%) (figure 1G).

Of the entire cohort, MRI brain and orbits were performed for 28/50 patients (56%), requested either by local teams or the paediatricians based at the study centre; 18 bilateral and 10 unilateral, with a combination of MAC phenotypes (7 microphthalmia, 1 anophthalmia, 6 coloboma and 14 mixed). Of the 22 patients not scanned, 13 had bilateral MAC while 9 were unilateral and 4 had syndromic MAC. Abnormal intracranial findings (including hypoplastic corpus callosum, hydrocephalus cerebellar hypoplasia and hemichiasm) were found in 7/28 patients (25%): Four were syndromic (34–1, 14–1, 24–1, 33–1) and three were non-syndromic (39–1, 42–1, 43–1) (figure 1G). Of these patients, six out of seven had bilateral MAC (86%), with only patient 43–1 presenting with unilateral simplex MAC.

Developmental delay/intellectual disability was found in eight patients, all of whom had an MRI: six bilateral and two unilateral. Patient 9–1 with Kabuki syndrome, 22–1 with learning difficulties and 22–1 with intellectual disability and autism all showed no intracranial abnormalities. Patients 35–1 and 35–2 were diagnosed with global developmental delay and severe intellectual disability, respectively, and both had bilateral sensorineural hearing loss, and 35–1 had cochlear implants fitted, however, neither had abnormal MRI findings.

All patients with kidney defects had bilateral MAC, with either microphthalmia (34–1, 35–2) or mixed MAC with microphthalmia and coloboma (24–1, 25–1). Patient 25–1 had right kidney scarring with recurrent urinary tract infections, while 24–1, 34–1 and 35–2 all had developmental delay alongside dysplastic kidneys, with hydronephrosis, multiple UTIs, and a duplex left kidney in patient 24–1, and cysts in 34–1 and 35–2.

### Molecular diagnosis

**Figure 2:**
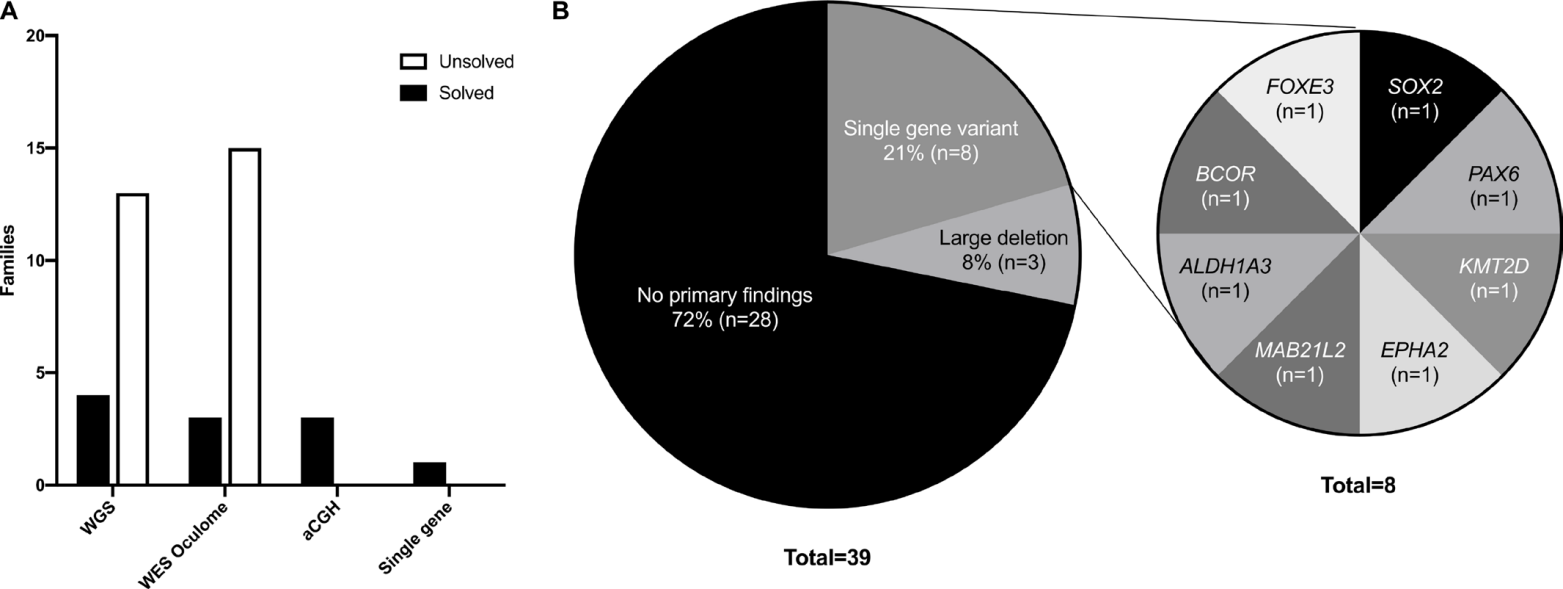
Molecular testing and results of microphthalmia, anophthalmia and coloboma (MAC) cohort. (a) Genetic testing and attainment of 39 families; (b) Molecular diagnoses of 39 families who underwent genetic tests, with monogenic pathogenic variants in 8 families. aCGH, microarray comparative genomic hybridisationa; CGH, microarray comparative genomic hybridization; WES, whole exome sequencing; WGS, whole genome sequencing.

Genetic testing was performed for 45/50 patients (90%) from 39/44 families (89%) by WGS (44%, 17/39), targeted gene panel (44%, 18/39), aCGH (8%, 3/39) or familial variant testing (5%, 1/39) (figure 2A). Of the five families where no genetic testing was performed: one family attended the genetic clinic prior to testing being available for MAC patients in 2017; one family declined testing; and three families did not attend their genetics appointments following referral. Patient 6–1 had previously undergone an aCGH prior to this study. Following genetic diagnosis of patient 35–2 from a targeted gene panel, patient 35–1 underwent a single gene test to confirm genetic cause. Patient 7–1 underwent a single gene test for *PAX6* variants, following previous familial diagnosis. Fifteen patients from 11 families obtained a genetic diagnosis (11/39, 28%), while 30 from 28 families had no primary findings. Family solve rate by test was 4/17 WGS (24%), 3/3 by aCGH (100%), 3/18 by targeted panel testing (17%) and 1/1 by familial variant testing only (figure 2A). Pathogenic variants were identified in eight genes: *SOX2*, *PAX6*, *KMT2D*, *EPHA2*, *MAB21L2*, *ALDH1A3*, *BCOR* and *FOXE3*, in addition to large deletions in chromosome 10, 11 and X.

Of those who underwent genetic testing, 10/30 bilateral and 5/15 unilateral patients had a genetic cause identified (table 2). Genetic diagnosis of unilateral cases was made using WGS (*MAB21L2*, *BCOR*), panel testing (*KMT2D*), single gene test (*PAX6*) and an aCGH (Chr10 deletion). Twenty-eight per cent (8/29) non-syndromic patients received a genetic diagnosis, compared with 44% (7/16) of syndromic patients. Using two-sided Fisher’s exact tests, no significant differences were found in solve rate by gender, laterality or presence of ocular/systemic features (table 2). The majority of solved patients had only microphthalmia (10/15), both anophthalmia patients were solved (2/2), no patients with an exclusive coloboma phenotype (0/9) and only 16% of the mixed cases with coloboma were solved (one anophthalmic and two microphthalmic, 3/19). However, sample sizes were too small to calculate Fisher’s exact test to look for significant differences in molecular diagnostic rates by MAC phenotype or ethnicity (>20% <5).

**Table 2:** Summary of phenotype data of microphthalmia, anophthalmia and coloboma (MAC) patients with molecular diagnostic testing

	Number of patients			Fisher’s exact test (p value)
	Total n=45	No primary findings n=30	Solved n=15	
Gender				
Male	16	10	6	0.75
Female	29	20	9	
Laterality				
Bilateral	30	20	10	>0.99
Unilateral	15	10	5	
MAC phenotype				
Microphthalmia	16	5	10	–
Anophthalmia	2	0	2	
Coloboma	9	9	0	
Mixed	18	16	3	
Ethnicity				
White (British)	17	10	7	–
Asian	10	5	5	
White (other background)	6	5	1	
Black (African)	1	1	0	
Unknown	11	9	2	
Additional ocular features				
Simplex	27	19	8	0.54
Complex	18	11	7	
Systemic involvement				
Non-syndromic	29	21	8	0.33
Syndromic	16	9	7	

All monogenic causes were previously associated with MAC except *EPHA2*, for which association with microphthalmia was validated and published,^19^ and novel variants were identified in MAC-associated genes *SOX2* c.867del;p.(Ser290Alafs*81), *KMT2D* c.6354del;p.(Ala2119Leufs*25), *MAB21L2* c.379A>T; p.(Lys127*), *ALDH1A3* c.104T>C;p.(Phe35Ser), *BCOR* c.856del;p.(Ser286Alafs*92) and *FOXE3* c.763dup;p.(Ala255Glyfs*30) (table 3, figure 2B).^19–27^ According to Association for Clinical Genomic Science classification guidelines, *ALDH1A3* variant c.104T>C;p.(Phe35Ser) in patient 22–1 is a variant of ‘uncertain significance’. However, the multidisciplinary team considered this patient’s phenotype to be compatible with reported *ALDH1A3* cases and there were no other relevant mutations found in MAC panels applied to this patient’s genome data. Prediction tools used classified the variant as probably damaging (Polyphen); damaging (SIFT); and deleterious (PredictSNP).

**Table 3:** Variant details for solved families of microphthalmia, anophthalmia and coloboma (MAC) patients

Family ID	Gene and transcript	OMIM#	Inheritance	Zygosity	Variant type	Variant	Pathogenicity /references	Ocular phenotype	Systemic features
4	Chr11 large deletion (including *YAP1*) NM_001282101	606 608	AD	Het	Large deletion including entire YAP1 gene	Large deletion	Heterozygous *YAP1* loss of function mutations previously reported to cause coloboma and microphthalmia phenotypes^34^	Bilateral microphthalmia/coloboma, retinal detachment	None
6	*SOX2* NM_003106	184 429	AD	Het	Frameshift deletion	c.867del; p.(Ser290Alafs*81)	Variant not previously reported. Absent from population databases. SIFT Indel neutral (0.787) Frameshift leading to prolonged protein.	Bilateral anophthalmia	Hypogonadism
7	*PAX6* NM_000280	607 108	AD	Het	Missense	c.372C>A; p.(Asn124Lys)	Previously reported^21^	Unilateral microphthalmia, aniridia, aphakia	DM type 2
9	*KMT2D* NM_003482	602 113	AD	Het	Frameshift deletion	c.6354del; p.(Ala2119Leufs*25)	Variant not previously reported. Absent from population databases. SIFT damaging (0.858)	Unilateral microphthalmia/coloboma	Kabuki syndrome, submucosal cleft palate
14	ChrX large deletion	–	XLD (mosaic)	Het (mosaic)	Mosaic large deletion	Xp22.2 - p11.1 deletion	–	Bilateral microphthalmia, corneal opacity	Linear skin defect syndrome, intellectual/developmental disability, abnormal intracranial findings) (hydrocephalous, agenesis of corpus callosum, cerebellar hypoplasia)
15	*EPHA2* NM_00431	176 946	AD	Het	Missense	c.1751C>T; p.(Pro584Leu)	Family subsequently reported^19^	Bilateral microphthalmia, congenital cataract, nystagmus, aphakia, glaucoma	None
16	*MAB21L2* NM_006439	604 357	AD	Het	Nonsense	c.379A>T; p.(Lys127*)	Variant not previously reported. Absent from population databases. PredictSNP deleterious (0.81)	Unilateral microphthalmia	None
22	*ALDH1A3* NM_000693	600 463	AR	Hom	Missense	c.104T>C; p.(Phe35Ser)	Variant not previously reported. Absent from population databases. Polyphen probably damaging (1.000) SIFT damaging (0) PredictSNP deleterious (0.87)	Bilateral anophthalmia	Autism spectrum, intellectual disability
28	*BCOR* NM_001123385	300 485	XLD	Het	Frameshift deletion	c.856del; p.(Ser286Alafs*92)	Variant not previously reported. Absent from population databases. SIFT Indel damaging (0.858)	Unilateral microphthalmia, congenital cataracts, aphakia, glaucoma	None
30	Chr10 deletion	–	AD	Het	Large deletion	Large deletion	–	Unilateral microphthalmia	None
35	*FOXE3* NM_012186	601 094	AR	Hom	Frameshift duplication	c.763dup; p.(Ala255Glyfs*30)	Variant not previously reported. Absent from population databases. SIFT Indel damaging (0.858)	Bilateral microphthalmia,	Bilateral enlarged kidneys with multiple cysts, bilateral sensorineural hearing loss, severe intellectual disability

Pathogenicity prediction from gnomAD (Genome Aggregation Database) for population frequencies (data set spans 125 748 exome sequences and 15 708 genome sequences from unique individuals)^15^; Polyphen2 (Polymorphism Phenotyping v2) predicts the possible impact of an amino acid substitution on the structure and function of a human protein (missense changes only) with a confidence score 0–1^16^; SIFT (Sorting Intolerant from Tolerant) predicts whether a non-synonymous amino acid substitution affects protein function, scored from 0 (deleterious) to 1 (tolerated)^17^; SIFT Indel classifies coding indels (insertion/deletions) with a confidence score 0–134^35^ ; PredictSNP (predictions for single nucleotide variants- that is, missense or nonsense) with a confidence score 0–1^18^. (https://www.omim.org/)

AD, autosomal dominant; AR, autosomal recessive; Chr, chromosome; DM, diabetes mellitus; Het, heterozygous; Hom, homozygous; OMIM, Online Mendelian Inheritance in Man; XLD, X-linked dominant.

## Discussion

The aetiology of MAC is complicated by a multitude of genetic and environmental factors resulting in highly variable phenotypes. The multiplex role of genes in regulating the development of ocular and extraocular tissues means that MAC is often associated with complex ocular phenotypes and systemic associations in 33%–95% of patients.^3 4^ Here, we report clinical presentation of 50 patients referred to the ocular genetic service at MEH. Paediatric and adult patients in this cohort were referred to the ocular genetic service at different stages of life, most having experienced various investigations and management by local teams. The care pathway provided by MEH enables a multidisciplinary approach from the outset, which incorporates full phenotyping (with ophthalmic and systemic examination including parents, appropriate imaging and paediatric review for children), visual and aesthetic rehabilitation, developmental support and genetic investigation.

Overall, lower BCVA in microphthalmic eyes compared with those with coloboma likely reflects the global effect on eye development, whereas visual impairment can vary depending on size and location of the colobomatous defect. It highlights the importance of determining the axial length through ultrasound B-scan or orbital MRI. Complex ocular features were found in 44% of patients, with cataracts occurring most frequently. An RD rate of 9% was identified in this cohort ranging from the first to the third decade; this varies widely in the literature (2.4%–42%) for patients with chorioretinal coloboma.^28 29^ Guidance regarding screening intervals and overall screening period is not clear and it is challenging to be prescriptive for rare and heterogeneous groups of patients with variable visual potentials. From this data, patients suitable for vitreoretinal surgery should be screened during their preverbal years and parents are given advice about monitoring for change in visual behaviour between intervals.

Heterogeneous MAC conditions in this study required a customised approach in prosthetic management, but general principles include assessment of ocular visual potential and the requirement for periorbital tissue rehabilitation before considering CCP treatment. Most patients (66%) did not require CCP and 7/50 were advised to have CCS as there was visual potential, but the eyes were deemed too small to support normal periocular development. It must be noted that some patients may still require socket surgery to support the use of a prosthesis or provide more symmetry between the periocular areas.

Seventeen patients (34%) had systemic involvement, in line with previous reports,^3 4^ and 56% had brain MRI scans, with intracranial abnormalities were found in 7/28, associated with intellectual disability/developmental delay in 3/7. The overwhelming majority of patients with abnormal intracranial findings had bilateral MAC (6/7, 86%) although previous reports have found no association with frequency/type of systemic abnormality by laterality.^30^ There is no definitive guidance on neuroimaging for MAC; however, this study reinforces the association with midline neurological defects including pituitary abnormalities and, hence, it is advisable to consider endocrinology work up and neuroimaging, particularly for bilateral cases.

Previous studies report higher molecular diagnostic rates for bilateral MAC patients (26–80%) than unilateral (10–20%).^4 7 31^ Molecular testing of 45/50 patients in the mixed MAC cohort yielded an overall diagnostic rate of 33%, which was consistent for both bilateral and unilateral cases, despite only 79% of unilateral cases having genetic testing compared with 97% of bilateral patients. Hence, both presentations warrant access to genetic testing as the rate of diagnosis is not preferentially higher for bilateral cases as previously considered. The highest proportion of molecular diagnoses were made from WGS (36%). A novel association of known cataract gene *EPHA2* with microphthalmia was identified,^19^ and the remaining monogenic causes had been previously associated with MAC, although with previously unreported pathogenic variants. Most genetic changes were in transcription factors (*SOX2, PAX6* and *FOXE3*)^10 21^ and molecules which regulate gene expression, such as histone modifier *KMT2D* and corepressor *BCOR*.^23 26 32^

A previously unreported association of *FOXE3* with bilateral sensorineural hearing loss was identified in two patients from family 35, alongside bilateral enlarged kidneys with multiple cysts. Biallelic pathogenic variants of forkhead transcription factor *FOXE3* are associated bilateral microphthalmia and coloboma, typically in non-syndromic patients; a recent study of 102 individuals found only 8% displayed extraocular features, including global developmental delay and autism.^10 27 31^ Systemic features were more commonly found in patients with truncating DNA changes; however, sensorineural hearing loss and kidney anomalies have not previously been reported. No other pathogenic variants were found from exome panel testing, and segregation of *FOXE3* with hearing loss in two patients suggests c.763dup; p.(Ala255Glyfs*30) is likely a recessive disease-causing variant affecting both eye and ear development; all parents were unaffected and found to be carriers. However, while the presence of an additional pathogenic mutation is unlikely it cannot be entirely disregarded.

We also report the first association of *MAB21L2* with unilateral MAC in a patient with non-syndromic, simplex microphthalmia and novel heterozygous nonsense variant, c.379A>T; p.(Lys127*). Pathogenic *MAB21L2* variants are found in patients with a spectrum of developmental ocular conditions, including bilateral MAC with or without systemic features, yet prior to this study, no unilateral MAC patients have been reported.^2 24 33^

Intrafamilial phenotypic variation was observed in MAC phenotype of family 20 and both MAC phenotype and additional ocular features of family 40. Patient 20–1 displayed bilateral chorioretinal colobomas with a microphthalmic right eye, while their affected sibling exhibited no microphthalmia, but bilateral iris and chorioretinal colobomas and an optic nerve coloboma in the right eye. Patient 20–2 also had myopia, while patient 20–1 had no non-MAC ocular conditions. Patient 40–1 had unilateral mixed MAC while their affected son exhibited bilateral MAC. Neither of these heterogeneous families obtained a genetic diagnosis, so the underlying cause of the variation in these cases cannot be yet established. However, a multitude of factors can lead to intrafamilial phenotypic variability, such as genetic modifiers, epigenetic variation and environmental factors influencing gene expression. These epistatic/external factors can make genetic diagnosis more difficult, as they can influence segregation patterns, and more work understanding and diagnosing these effects would provide further insight into MAC aetiology.

Patients diagnosed with a molecular cause were directed to appropriate specialists for investigation and management of ocular/systemic features where genotype–phenotype correlations were known. Furthermore, genetically solved patients in this cohort were given access to genetic counselling to provide information on the potential impacts of their test results and guide future choices. However, with less than a third of families (28%) obtaining a genetic diagnosis, unfortunately many families could not be given specific clinical care or advice based on their underlying molecular cause, resulting in greater uncertainty regarding prognosis and family planning.

Clear understanding of MAC aetiology is key to providing appropriate clinical care and genetic counselling. However, genetic heterogeneity and complex inheritance patterns make molecular diagnosis challenging, particularly for unilateral cohorts. This work highlights the importance of careful phenotyping, to assemble the appropriate multidisciplinary care team to undertake investigations such as MRI brain imaging and genetic testing. Increased usage of next-generation sequencing to identify novel variants and complex non-mendelian causes of MAC can clarify genotype–phenotype relationships, pointing to potential comorbidities, which ensure that patients are referred to the correct specialists, which can improve prognosis.

## Data Availability

All data relevant to the study are included in the article or uploaded as supplementary information.

## References

[1] Harding P , Moosajee M . The molecular basis of human anophthalmia and microphthalmia. J Dev Biol 2019;7:16. 10.3390/jdb7030016 31416264 PMC6787759

[2] Deml B , Kariminejad A , Borujerdi RHR , et al . Mutations in MAB21L2 result in ocular coloboma, microcornea and cataracts. PLoS Genet 2015;11:e1005002–26. 10.1371/journal.pgen.1005002 25719200 PMC4342166

[3] Slavotinek A . Genetics of anophthalmia and microphthalmia. Part 2: syndromes associated with anophthalmia–microphthalmia. Hum Genet 2018:1–16. 10.1007/s00439-018-1949-1 30374660

[4] Shah SP , Taylor AE , Sowden JC , et al . Anophthalmos, microphthalmos, and coloboma in the United Kingdom: clinical features, results of investigations, and early management. Ophthalmology 2012;119:362–8. 10.1016/j.ophtha.2011.07.039 22054996

[5] Källén B , Tornqvist K . The epidemiology of anophthalmia and microphthalmia in Sweden. Eur J Epidemiol 2005;20:345–50. 10.1007/s10654-004-6880-1 15971507

[6] Hornby SJ , Gilbert CE , Rahi JK , et al . Regional variation in blindness in children due to microphthalmos, anophthalmos and coloboma. Ophthalmic Epidemiol 2000;7:127–38. 10934463

[7] Plaisancie J , Calvas P , Chassaing N . Genetic advances in microphthalmia. J Pediatr Genet 2016;5:184–8. 10.1055/s-0036-1592350 27895970 PMC5123893

[8] Patel A , Hayward JD , Tailor V , et al . The oculome panel test: next-generation sequencing to diagnose a diverse range of genetic developmental eye disorders. Ophthalmology 2019;126:888–907. 10.1016/j.ophtha.2018.12.050 30653986

[9] Schilter KF , Reis LM , Schneider A , et al . Whole-genome copy number variation analysis in anophthalmia and microphthalmia. Clin Genet 2013;84:473–81. 10.1111/cge.12202 23701296 PMC3985344

[10] Williamson KA , FitzPatrick DR . The genetic architecture of microphthalmia, anophthalmia and coloboma. Eur J Med Genet 2014;57:369–80. 10.1016/j.ejmg.2014.05.002 24859618

[11] Turnbull C , Scott RH , Thomas E , et al . The 100 000 genomes project: bringing whole genome sequencing to the NHS. BMJ 2018;361:k1687. 10.1136/bmj.k1687 29691228

[12] Schiff ER , Tailor VK , Chan HW , et al . Novel biallelic variants and phenotypic features in patients with SLC38A8-related foveal hypoplasia. Int J Mol Sci 2021;22:1130. 10.3390/ijms22031130 33498813 PMC7866073

[13] Stenson PD , Mort M , Ball EV , et al . The human gene mutation database: building a comprehensive mutation repository for clinical and molecular genetics, diagnostic testing and personalized genomic medicine. Hum Genet 2014;133:1–9. 10.1007/s00439-013-1358-4 24077912 PMC3898141

[14] Landrum MJ , Lee JM , Benson M , et al . ClinVar: improving access to variant interpretations and supporting evidence. Nucleic Acids Res 2018;46:D1062–7. 10.1093/nar/gkx1153 29165669 PMC5753237

[15] Karczewski KJ , Francioli LC , Tiao G , et al . The mutational constraint spectrum quantified from variation in 141,456 humans. Nature 2020;581:434–43. 10.1038/s41586-020-2308-7 32461654 PMC7334197

[16] Adzhubei I , Jordan DM , Sunyaev SR . Predicting functional effect of human missense mutations using PolyPhen-2. Curr Protoc Hum Genet 2013;Chapter 7:1–52. 10.1002/0471142905.hg0720s76 PMC4480630 23315928

[17] Sim N-L , Kumar P , Hu J , et al . SIFT web server: predicting effects of amino acid substitutions on proteins. Nucleic Acids Res 2012;40:W452–7. 10.1093/nar/gks539 22689647 PMC3394338

[18] Bendl J , Stourac J , Salanda O , et al . PredictSNP: robust and accurate consensus classifier for prediction of disease-related mutations. PLoS Comput Biol 2014;10:e1003440. 10.1371/journal.pcbi.1003440 24453961 PMC3894168

[19] Harding P , Toms M , Schiff E , et al . EPHA2 segregates with microphthalmia and congenital cataracts in two unrelated families. Int J Mol Sci 2021;22:2190. 10.3390/ijms22042190 33671840 PMC7926380

[20] Schneider A , Bardakjian T , Reis LM , et al . Novel SOX2 mutations and genotype-phenotype correlation in anophthalmia and microphthalmia. Am J Med Genet A 2009;149A:2706–15. 10.1002/ajmg.a.33098 19921648 PMC2787970

[21] Williamson KA , Hall HN , Owen LJ . Recurrent heterozygous Pax6 missense variants cause severe bilateral microphthalmia via predictable effects on DNA–protein interaction. Genet Med 2019. 10.1038/s41436-019-0685-9 PMC7056646 31700164

[22] Cuvertino S , Hartill V , Colyer A , et al . A restricted spectrum of missense KMT2D variants cause a multiple malformations disorder distinct from kabuki syndrome. Genet Med 2020;22:867–77. 10.1038/s41436-019-0743-3 31949313 PMC7200597

[23] Bögershausen N , Altunoglu U , Beleggia F , et al . An unusual presentation of kabuki syndrome with orbital cysts, microphthalmia, and cholestasis with bile duct paucity. Am J Med Genet A 2016;170:3282–8. 10.1002/ajmg.a.37931 27530281

[24] Rainger J , Pehlivan D , Johansson S , et al . Monoallelic and biallelic mutations in MAB21L2 cause a spectrum of major eye malformations. Am J Hum Genet 2014;94:915–23. 10.1016/j.ajhg.2014.05.005 24906020 PMC4121478

[25] Fares-Taie L , Gerber S , Chassaing N , et al . ALDH1A3 mutations cause recessive anophthalmia and microphthalmia. Am J Hum Genet 2013;92:265–70. 10.1016/j.ajhg.2012.12.003 23312594 PMC3567280

[26] Ragge N , Isidor B , Bitoun P , et al . Expanding the phenotype of the X-linked BCOR microphthalmia syndromes. Hum Genet 2019;138:1051–69. 10.1007/s00439-018-1896-x 29974297

[27] Reis LM , Sorokina EA , Dudakova L , et al . Comprehensive phenotypic and functional analysis of dominant and recessive FOXE3 alleles in ocular developmental disorders. Hum Mol Genet 2021;30:1591–606. 10.1093/hmg/ddab142 34046667 PMC8369840

[28] Suwal B , Paudyal G , Thapa R , et al . A study on pattern of retinal detachment in patients with choroidal coloboma and its outcome after surgery at a tertiary eye hospital in Nepal. J Ophthalmol 2019;2019:1–5. 10.1155/2019/7390852 PMC6501416 31143474

[29] Hussain RM , Abbey AM , Shah AR , et al . Chorioretinal coloboma complications: retinal detachment and choroidal neovascular membrane. J Ophthalmic Vis Res 2017;12:3–10. 10.4103/2008-322X.200163 28299000 PMC5340060

[30] Skalicky SE , White AJR , Grigg JR , et al . Microphthalmia, anophthalmia, and coloboma and associated ocular and systemic features: understanding the spectrum. JAMA Ophthalmol 2013;131:1517–24. 10.1001/jamaophthalmol.2013.5305 24177921

[31] Chassaing N , Causse A , Vigouroux A , et al . Molecular findings and clinical data in a cohort of 150 patients with anophthalmia/microphthalmia. Clin Genet 2014;86:326–34. 10.1111/cge.12275 24033328

[32] Froimchuk E , Jang Y , Ge K . Histone H3 lysine 4 methyltransferase KMT2D. Gene 2017;627:337–42. 10.1016/j.gene.2017.06.056 28669924 PMC5546304

[33] Horn D , Prescott T , Houge G , et al . A novel oculo-skeletal syndrome with intellectual disability caused by a particular MAB21L2 mutation. Eur J Med Genet 2015;58:387–91. 10.1016/j.ejmg.2015.06.003 26116559

[34] Williamson KA , Rainger J , Floyd JAB , et al . Heterozygous loss-of-function mutations in YAP1 cause both isolated and syndromic optic fissure closure defects. Am J Hum Genet 2014;94:295–302. 10.1016/j.ajhg.2014.01.001 24462371 PMC3928658

[35] Hu J , Ng PC . SIFT indel: predictions for the functional effects of amino acid insertions/deletions in proteins. PLoS One 2013;8:e77940. 10.1371/journal.pone.0077940 24194902 PMC3806772

